# Value of ^18^F-FDG PET/CT for Assessment of Advanced Lacrimal Sac Non-Keratinizing Squamous Cell Carcinoma Successfully Treated with Concurrent Chemoradiotherapy

**DOI:** 10.3390/diagnostics11111961

**Published:** 2021-10-22

**Authors:** Ching-Yu Liao, Li-An Huang, Yu-Hsuan Lin

**Affiliations:** 1 Department of Medical Education, Chang-Geng Medical Foundation Linkou Chang-Geng Memorial Hospital, Taoyuan 131, Taiwan; mxi346@gmail.com; 2 Department of Medical Education, Chung Shan Medical University Hospital, Taichung 402, Taiwan; lian8581@gmail.com; 3 Institute of Biomedical Sciences, National Sun Yat-Sen University, Kaohsiung 804, Taiwan; 4 Department of Otolaryngology, Head and Neck Surgery, Kaohsiung Veterans General Hospital, Kaohsiung 813, Taiwan; 5 School of Medicine, National Yang Ming Chiao Tung University, Taipei 112, Taiwan; 6 School of Medicine, Chung Shan Medical University, Taichung 402, Taiwan

**Keywords:** ^18^F-FDG PET/CT, NKSCC, concurrent chemo-radiotherapy, lacrimal sac

## Abstract

Non-keratinizing squamous cell carcinoma (NKSCC) of the lacrimal apparatus is extremely rare. It is usually very aggressive in destroying local tissue and has a grave prognosis for relentless recurrence and distant failures. Though the current evidence cannot make confident recommendations regarding the best management, curative surgical excision with adjuvant radiotherapy remains the most commonly used strategy. Here, we report a 71-year-old woman presented with progressive right medial canthal swellings for six months. A transnasal endoscopic biopsy revealed NKSCC of the lacrimal sac. She then underwent a combination of magnetic resonance images (MRI) and 2-deoxy-2-(18F)fluoro-D-glucose positron emission tomography/computed tomography (^18^F-FDG PET/CT) for staging purposes. Following cisplatin-based concurrent chemo-radiotherapy (CCRT), the post-treatment PET/CT illustrated the absence of an abnormal metabolic accumulation over the suspicious region as observed in post-treatment CT. A further trans-ostia re-biopsy confirmed complete tumor remission. This case demonstrates the remarkable ability of ^18^F-FDG PET/CT to differentiate between a persistent malignancy and post-treatment changes. Furthermore, a definite CCRT might provide comparable outcomes to traditional surgery.

**Figure 1:**
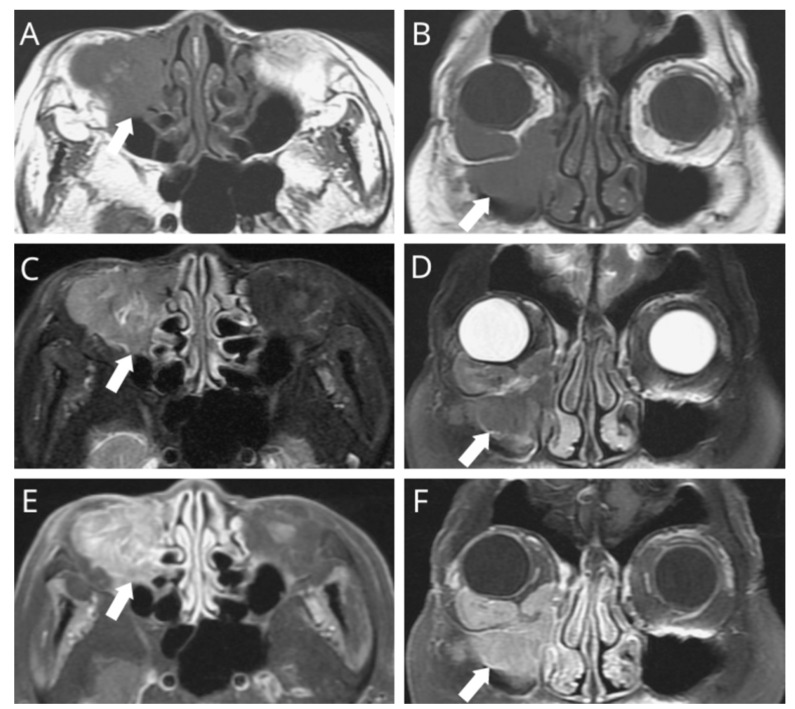
A 71-year-old woman visited our clinic due to right medial orbital swellings associated with limited ocular mobility, drooping eyelids, blurred vision, and persistent tearing in the previous six months. On further ophthalmic testing, there was a grade-3 infraversion oculus dexter, but a naso-endoscopy demonstrated negative findings. ((**A**,**B**) arrow denotes the primary tumor) A subsequent MRI revealed a lesion of irregular contour presenting with homogenous hypointense signals on the T1-weighted sequence, with an associated tumor growth measuring 4.2 cm. ((**C**,**D**) arrow denotes the primary tumor) The sagittal and coronal fat-saturated T2-weighted sequence revealed the lesion location within the lacrimal sac, extending into the right maxillary sinus and orbit, causing prominent bony destruction. ((**E**,**F**) arrow denotes the primary tumor) The fat-saturated T1-weighted MRI found a vivid heterogeneous enhancement upon contrast administration.

**Figure 2:**
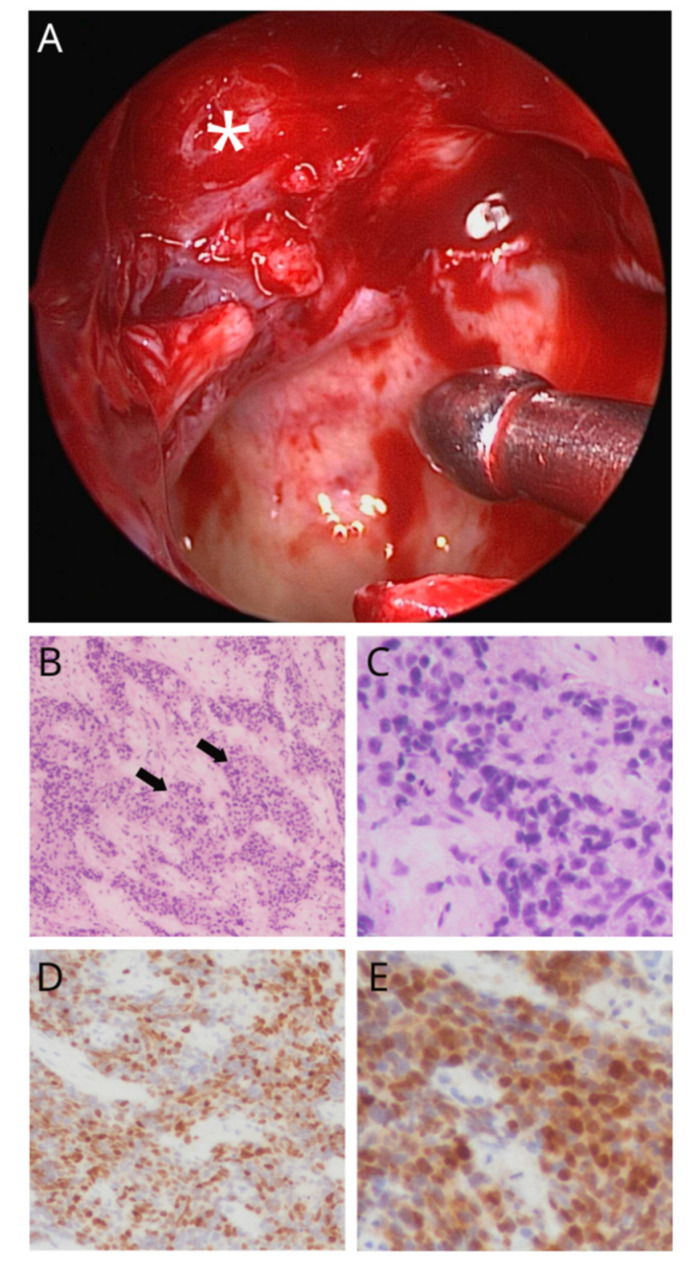
Specimen retrieved via endonasal endoscopic surgery. ((**A**) asterisk) The intra-operative findings demonstrated that the tumor located on the superior aspect of the sinus bled easily. Hematoxylin-eosin staining demonstrated ((**B**) 100×; arrow denotes the nesting arrangement) a nesting arrangement and ((**C**) 400×) occasional anastomoses with pleomorphic nuclei and prominent nucleoli. The immunohistochemistry found tumor cells positive for p40 ((**D**) 200×) and p63 ((**E**) 400×) and negative for p16 and NUT. INI-1 and SMARCA4 expressed no aberrant loss. Taken together, the pathological diagnosis of non-keratinizing squamous cell carcinoma (NKSCC) was made.

**Figure 3:**
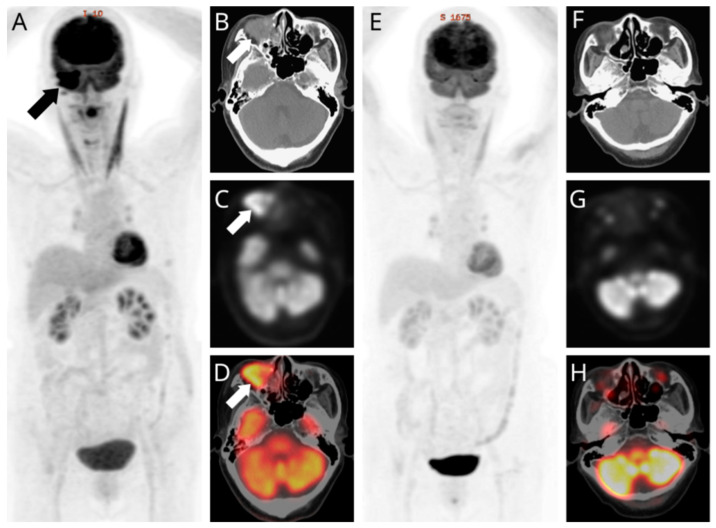
Selected whole-body ^18^F-FDG PET/CT images (maximal intensity projection [MIP]: (**A**,**E**); CT: (**B**,**F**); PET: (**C**,**G**); fused PET/CT: (**D**,**H**); lesion annotated by arrows). (**D**) We identified abnormal ^18^F-FDG accumulations within the lacrimal apparatus, paranasal sinus, and extra-conal orbital compartment, with a maximal standardized uptake value of 11.5. (**A**) As the MIP demonstrated no evidence of regional lymph nodes or distant organ involvement, the patient was classified as American Joint Committee on Cancer T4N0M0, stage IV. She declined surgical intervention, electing cisplatin-based concurrent chemoradiotherapy (CCRT) as her definite treatment strategy. The resolution of local orbital swellings and neurologic deficits was noted post-treatment. A three-month follow-up computed tomography was arranged to assess the tumor response, (**F**) revealing a suspicious soft-tissue density remnant over the inferior orbit. However, there were no abnormal accumulations in (**E**) the post-CCRT MIP, and (**H**) the corresponding ^18^F-FDG PET/CT revealed negative evidence for a recurrent or persistent disease, supported by the negative histopathologic findings of the suspicious region through a re-biopsy. NKSCC, previously known as transitional cell carcinoma due to its composition of irregular stratified epithelium and papillary growth pattern [1,2], is a rare histology entity for primary malignant lacrimal drainage systems, with less than 50 described in the previous literature [2]. Lacrimal sac NKSCC typically manifests around the fifth decade of life and without gender predilection [3]. Due to its hidden anatomic location, lacrimal sac NKSCC usually presents with an insidious course and vague symptoms, thereby generating difficulties in early-stage diagnosis [4]. At present, a wide resection followed by adjuvant radiotherapy is the most utilized strategy for lacrimal sac NKSCC [2,5], but the prognosis remains unfavorable, with an estimated overall survival rate of roughly 50%. Most cases succumb to distant metastasis and/or the late detection of local recurrence [6,7]. On the other hand, the efficacy of primary radiotherapy is unclear because of the paucity of evidence [8]. Despite refusing surgical intervention and electing CCRT, our patient maintained a disease-free status for 23 months on follow up. To conclude, this case, therefore, highlights the high clinical value of ^18^F-FDG PET/CT for the initial staging and post-treatment evaluation of therapeutic responses when traditional images demonstrate equivocal findings [9,10]. Additionally, we illustrate how cisplatin-based CCRT may act as an effective alternative to surgical resections.

## Data Availability

The data presented in this study are available on request from the corresponding author.
